# Adverse Life Trajectories Are a Risk Factor for SARS-CoV-2 IgA Seropositivity

**DOI:** 10.3390/jcm10102159

**Published:** 2021-05-17

**Authors:** Cyrielle Holuka, Chantal J. Snoeck, Sophie B. Mériaux, Markus Ollert, Rejko Krüger, Jonathan D. Turner, the CON-VINCE Consortium

**Affiliations:** 1 Immune Endocrine Epigenetics Research Group, Department of Infection and Immunity, Luxembourg Institute of Health, L-4354 Esch-sur-Alzette, Luxembourg; cyrielle.holuka@lih.lu (C.H.); Sophie.Meriaux@lih.lu (S.B.M.); 2 Faculty of Science, University of Luxembourg, L-4365 Belval, Luxembourg; 3 Clinical and Applied Virology Group, Department of Infection and Immunity, Luxembourg Institute of Health, L-4354 Esch-sur-Alzette, Luxembourg; chantal.snoeck@lih.lu; 4 Allergy and Clinical Immunology, Department of Infection and Immunity, Luxembourg Institute of Health, 29, rue Henri Koch, L-4354 Esch-sur-Alzette, Luxembourg; markus.ollert@lih.lu; 5 Odense Research Center for Anaphylaxis, Department of Dermatology and Allergy Center, University of Southern Denmark, 5000 Odense, Denmark; 6 Transversal Translational Medicine, Luxembourg Institute of Health (LIH), L-1445 Strassen, Luxembourg; rejko.krueger@lih.lu; 7 Luxembourg Centre for Systems Biomedicine, University of Luxembourg, L-4362 Esch-Sur-Alzette, Luxembourg

**Keywords:** SARS-CoV-2, COVID-19, early-life adversity, adult traumatic events, psychosocial adversity, relative risk, serology

## Abstract

Asymptomatic individuals, called “silent spreaders” spread SARS-CoV-2 efficiently and have complicated control of the ongoing COVID-19 pandemic. As seen in previous influenza pandemics, socioeconomic and life-trajectory factors are important in disease progression and outcome. The demographics of the asymptomatic SARS-CoV-2 carriers are unknown. We used the CON-VINCE cohort of healthy, asymptomatic, and oligosymptomatic individuals that is statistically representative of the overall population of Luxembourg for age, gender, and residency to characterise this population. Gender (male), not smoking, and exposure to early-life or adult traumatic experiences increased the risk of IgA seropositivity, and the risk associated with early-life exposure was a dose-dependent metric, while some other known comorbidities of active COVID-19 do not impact it. As prior exposure to adversity is associated with negative psychobiological reactions to external stressors, we recorded psychological wellbeing during the study period. Exposure to traumatic events or concurrent autoimmune or rheumatic disease were associated with a worse evolution of anxiety and depressive symptoms throughout the lockdown period. The unique demographic profile of the “silent spreaders” highlights the role that the early-life period plays in determining our lifelong health trajectory and provides evidence that the developmental origins of health and disease is applicable to infectious diseases.

## 1. Introduction

First reports of an outbreak of a novel coronavirus disease (COVID-19) in Wuhan, China, appeared in December 2019. This was rapidly attributed to a betacoronavirus principally affecting the respiratory system, severe acute respiratory syndrome coronavirus 2 (SARS-CoV-2) [1]. This rapidly escalated, reaching the pandemic level in March 2020 [2]. As of now, more than 151 million cases of COVID-19 have been reported, and over 3 million deaths recorded worldwide [3]. COVID-19 symptoms appear between 2 and 14 days after exposure. However, many SARS-CoV-2-infected individuals display no or only mild symptoms [4,5,6,7,8] even though they develop a clear, but weaker immune response to the virus than other COVID-19 patients [9]. It has become clear that there are many inequalities in severity and susceptibility to COVID-19 [10]. In a manner reminiscent of the influenza pandemics of 1918 and 2009 [11,12,13], initial data suggest that lower socioeconomic status (SES) is associated with increased mortality from COVID-19 [14], and this was observed in cohorts from the USA, the UK, and China. However, we currently know very little about the demographics of the asymptomatic SARS-CoV-2 carriers.

The overall life trajectory from conception, through early life, and towards adulthood plays a preponderant role in determining the risk of a wide range of non-communicable diseases including mental health, diabetes, cardiovascular disease, and obesity [15,16]. This produced the theory of the developmental origins of health and disease (DOHaD) [17]. This theory has been subsequently refined and can now be thought of as a “three hit model”, incorporating genetic predisposition (hit one) and environmental insults during a sensitive period (hit 2). This produces a latent, quiescent phenotype. Many years later the risk is crystallised by a third hit in the later life environment [18,19]. The early-life period appears to be particularly sensitive to the external environment, with effects acting over many decades [17,20,21].

Although early-life adversity (ELA) covers an almost infinite range of stressors, psychosocial stress is predominant [22,23]. In the adverse childhood experiences (ACE) study [24], more than 50% of the study participants had experienced one (or more) forms of ELA, and 12% had experienced more than four forms of ELA. In the context of a viral pandemic this is particularly important, as ELA not only induces a clear, lifelong, pro-inflammatory immunophenotype [25,26], but also induces significant changes in antiviral cytotoxic T-cells (CTLs, CD8+ T-cells), rendering them largely senescent and reducing their cytotoxicity [27,28,29,30]. Consequently, ELA-exposed populations may have a higher risk of viral respiratory episodes compared to the general population. In the context of identifying exposed populations, it is therefore logical to include a history of traumatic life events.

In the case of infections that are asymptomatic or oligosymptomatic IgA is the most suitable immunoglobulin to analyse. In such mild cases, infection is limited to the upper respiratory tract where IgA is the predominant Ig [31] as the primary immune response originates from the mucosal immune system, particularly from the nasopharynx-associated lymphoid tissue (NALT) [32]. This parallels what is seen in HIV infection, where a mucosal IgA response is completely protective [33], something that is also seen for *Picornaviridae* (e.g., poliovirus), *Reoviridae* (e.g., rotavirus), or *Adenoviridae* and is essential in all cases to avoid infection [33,34,35,36].

There is now growing evidence that asymptomatic individuals can spread SARS-CoV-2 efficiently. The presence of these “silent spreaders” has complicated the control of the pandemic [4,6]. In this study, we describe the epidemiological, demographic, socioeconomic, and prior psychosocial life trajectory (including a history of ELA) in asymptomatic individuals within a cohort that is statistically representative of the entire Luxembourgish population. Furthermore, ELA has a major impact on mental health [37]. The CON-VINCE cohort of asymptomatic carriers, with a fixed disease severity, allowed us to examine both the socioeconomic factors underlying exposure to SARS-CoV-2, as well as factors that predispose individuals to a mild disease course, and the role that a negative life trajectory played in the subsequent response to the lockdown.

## 2. Materials and Methods

### 2.1. Cohort

This study used the previously reported CON-VINCE cohort [2]. Briefly, the CON-VINCE cohort is representative for the overall Luxemburgish adult population for age, sex, and residency [2]. All participants (*n* = 1862) were recruited between 15 April and 5 May 2020 and underwent bi-weekly blood and pooled nasal and oropharyngeal swab sampling for 10 weeks, until 26 June 2020. SARS-CoV-2 rRT-PCR together with IgA and IgG serology was performed at each bi-weekly sampling point together with a series of online questionnaires [2]. Inclusion criteria included people aged 18 and over and capable of providing informed consent that, at inclusion, were either (i) SARS-CoV-2 negative, (ii) SARS-CoV-2 positive but asymptomatic or oligosymptomatic (i.e., no fever, respiratory distress or cough not attributable to a pre-existing comorbidity), or (iii) post-infectious SARS-CoV-2 negative after a mild disease course. The CON-VINCE study was approved by the Comité National d’Ethique de Recherche (CNER, reference 202004/01) and the Ministry of Health (Luxembourg, reference 831x6ce0d), and is registered on ClinicalTrials.gov (NCT 04379297, accessed on 10 March 2021).

### 2.2. Data Collection

As previously reported [2] participants provided (i) demographic data including age, gender, origin, marital status, household composition, (ii) medical history, and (iii) socioeconomic data including educational attainment, employment status and category, annual income, and home-ownership through an online reporting system at inclusion.

### 2.3. Psychological Questionnaires

The baseline and bi-weekly follow-up questionnaires addressed the current medical and psychological states of the participants during the study period. As previously described, participants completed four psychological questionnaires: Center for Epidemiologic Studies Depression Scale (CES-D), Generalised Anxiety Disorder-7 (GAD-7), Perceived Social Stress (PSS), and UCLA loneliness scale (UCLA) in their unmodified forms [38,39,40,41]. In the final follow-up questionnaire, ELA exposure was measured with the 28-item Childhood Trauma Questionnaire (CTQ) [42] retrospectively assessing physical, emotional, and sexual abuse as well as physical and emotional neglect during childhood. The overall CTQ score (scale 0–4), and the five subscales were calculated as previously described [42]. Question replies were scored from 0–4 (never true, rarely true, sometimes true, often true, very often true). The three validity items (10, 16, 22) and questions 2, 3, 5, 7, 13, 19, 22, 26, and 28 were reverse scored (i.e., Q16 “I thought I had a perfect childhood”: Very true = 0). The overall score and the three subscale scores were calculated as described by Bernstein et al. [42]. The mean of the three validity scores was compared to the overall CTQ score. Participants with differences ≥1.5 units difference between the overall and validity score were excluded from the dataset.

Exposure to traumatic events and principal psychosocial stressors in adulthood including death of a family member, job loss, financial difficulties, or divorce was assessed using the questions previously reported [43]. This questionnaire includes the salience (interpretation) of the event by the individual. A positive salience is exposure to a trauma e.g., divorce, but the overall experience was positive (such as escaping from a poor marriage), and a negative salience is undergoing the same event, but experiencing it is a negative, traumatic manner (such as the divorce being surprising and imposed).

### 2.4. IgA and IgG Serology 

As previously reported [2], IgA and IgG levels specific to SARS-CoV-2 were determined by ELISA (Anti-SARS-CoV-2 ELISA IgA; Anti-SARS-CoV-2 ELISA IgG; Euroimmun, Lübeck, Germany) according to the manufacturer’s instructions. Samples were considered positive with an OD ratio ≥1.1, borderline OD ratios (>0.8, <1.1) were considered positive for further analyses, and samples were considered negative with an OD ratio <0.8. 

### 2.5. Statistical Analyses and Data Presentation 

All data analyses were performed in R (version 3.6.3 R Core Team, 2019) running in R studio (version 1.3.959; R Core Team, 2019). Data cleaning, sorting, and dichotomisation was performed with the packages dplyr [44] and sjmisc [45]. Uncorrected relative risks were calculated for individual covariates independently from contingency tables using the package EpiTools [46]. Corrected relative risks were calculated using R base functions through general logistic regression models. Initially, sex and smoking (univariate *p* < 0.05) were included as covariates in the corrected RR models. As sex was the only covariate that was significant in the adjusted RR model, the calculations were repeated and the data reported only for the sex-adjusted RR. Relative risks were subsequently extracted from the logistic regression model using the package epiDisplay [47]. K-means clustering of the Adult Traumatic Events questionnaire was performed using the package cluster [48]. In all analyses the covariates were included as explanatory variables for the IgA seropositivity outcome variable, and comparisons between covariates were not assessed. Figures were subsequently generated using SigmaPlot (version 12.5) and Adobe Illustrator CS6 (version 16.00). Data and statistical scripts are available upon reasonable request.

## 3. Results

### 3.1. Demographic Covariates 

**Figure 1:**
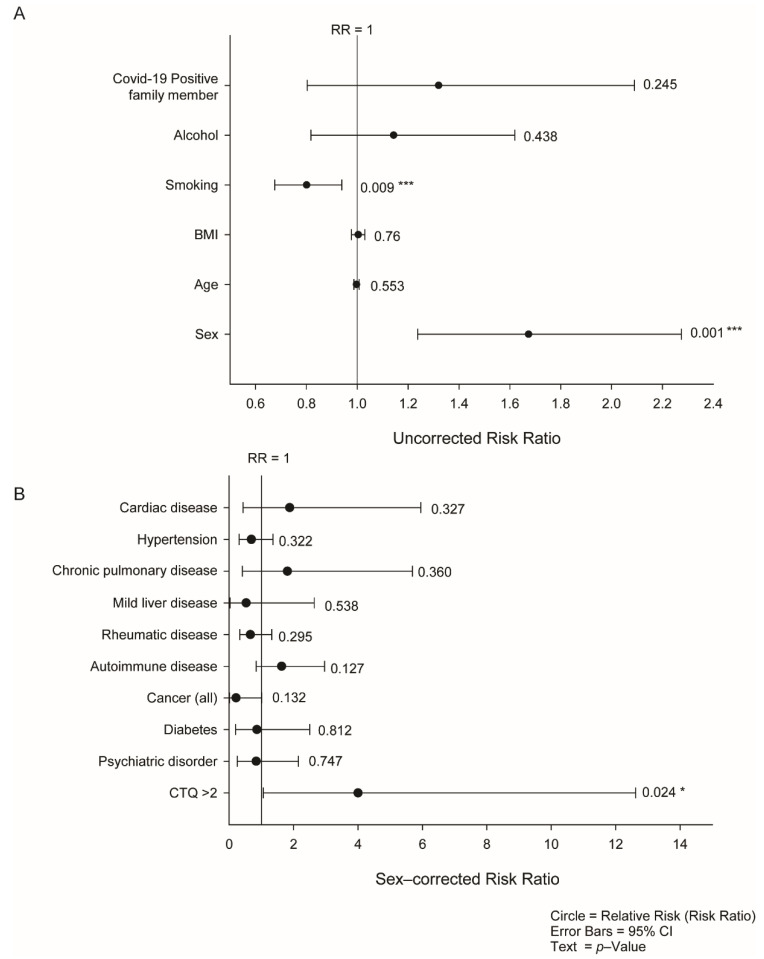
Demographics of SARS-CoV-2 IgA seropositivity in asymptomatic or oligosymptomatic individuals. (**A**) Crude relative risk estimates for SARS-CoV-2 IgA seropositivity for all members of the CON-VINCE cohort finishing the five experimental visits. (**B**) Sex-adjusted logistic regression relative risk of being SARS-CoV-2 IgA seropositive due to pre-existing comorbidities. Both panels: Circles represent the relative risk (RR); error bars: 95% confidence interval; text: *p*-value. *, *p* < 0.05; ***, *p* < 0.005.

The CON-VINCE cohort recruited 1862 participants, and 1537 participants completed the study. Anti-SARS-CoV-2 IgA and IgG serology, together with complete medical, demographic, socioeconomic, lifestyle, and traumatic data were available for 1418 participants. As previously reported, this is above the threshold for a statistically representative sample of the entire Luxemburgish population [2]. In total, 199 participants had at any one point during the study a positive IgA serology test. IgG seroprevalence was significantly lower with only 41 participants having a positive serology result at any one point in the 12 weeks of the study, which is partly explained by the natural sequence of immune response to a recent exposure. Prior to calculating the relative risk of anti-SARS-CoV-2 IgA or IgG seropositivity associated with pre-existing comorbidities, or psychosocial and lifestyle parameters, we initially calculated crude uncorrected relative risks (RR) for the common demographic covariates, i.e., age, alcohol consumption, sex, smoking, and BMI (Figure 1A). RR were initially calculated for both IgA and IgG seropositivity. No significant associations were observed with IgG seropositivity due to the low number of positive participants. Subsequently, IgA seropositivity was used exclusively throughout the study. There were two significant demographic covariates that were associated with IgA seropositivity: sex (males) and smoking (*p* = 0.001 and 0.009 respectively). Having a family member with COVID-19 increased the relative risk 1.3-fold, but was not significant (*p* > 0.1). In the CON-VINCE cohort, age, BMI, and alcohol consumption were not significantly associated with IgA seropositivity. Categorical breakdowns of these key demographic covariates are provided in Table 1. 

Table 1 shows the covariates of age, BMI, and alcohol consumption associated with the IgA seropositivity percentage for categorical breakdowns.

### 3.2. Relative Risk Linked to Pre-Existing Comorbidities

The number of participants with each of the participant-reported comorbidities are given in Table 2. To examine the role of the pre-existing comorbidities we calculated sex, smoking, and having a COVID-positive household member adjusted RR models when the incidence of the comorbidity was >1% of the cohort (>14 participants with a certain comorbidity). In none of the models were smoking or having a COVID-positive household member significant covariates (*p* > 0.1), and neither had a significant interaction term between them or with sex. As such, smoking or having a COVID-positive household member were dropped from all reported models. None of the pre-existing comorbidities increased the RR significantly (Figure 1B). However, participants with a history of malignant disease (any type) had a non-significantly lower risk of IgA seropositivity (RR = 0.215; 95% CI from 0.012 to 1.01; *p* = 0.012). As ELA can directly impact the immune system, we calculated the RR when CTQ > 2. The risk of being IgA seropositive increased with exposure to ELA (RR = 4.00, 95% CI: 1.06 to 12.62, *p* = 0.024) (Figure 1B).

Table 2 shows exact number of participants per comorbidities with the ratio of female/male. The *p*-value and percentage of IgA positivity are given for each comorbidity.

### 3.3. Early-Life Adversity Incidence and the Associated Increases in Risk of IgA Seropositivity 

**Figure 2:**
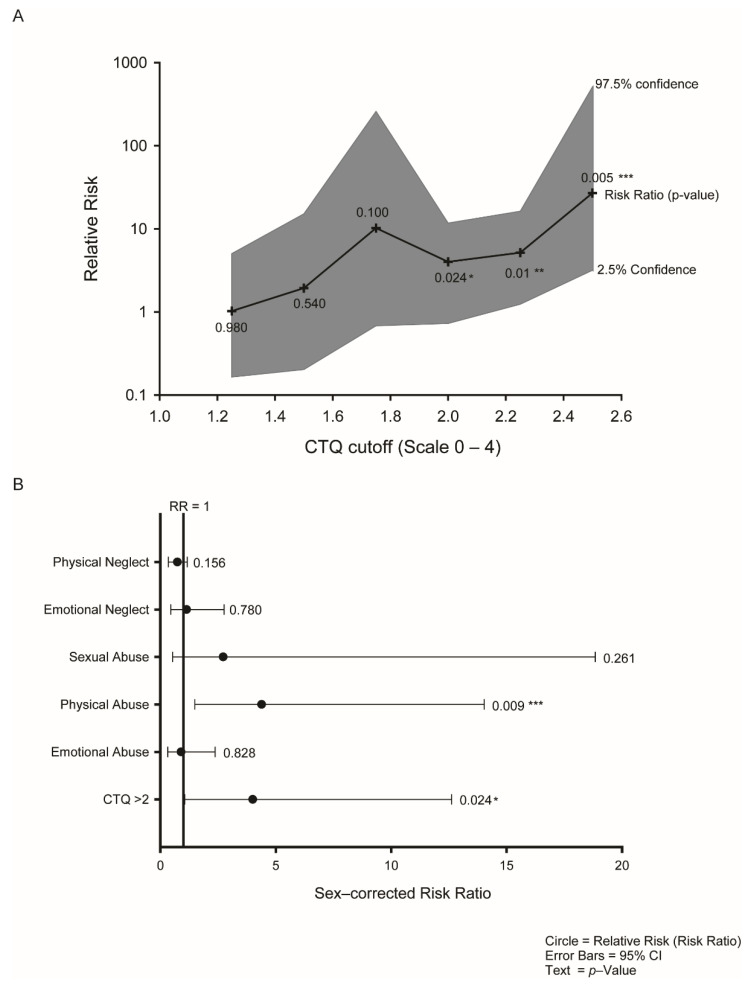
Exposure to early-life adversity is associated with SARS-CoV-2 seropositivity in asymptomatic or oligosymptomatic individuals. (**A**) ELA is linked to SARS-CoV-2 seropositivity in a dose-dependent manner as the cutoff for being considered subject to ELA increases. The central black line represents the sex-adjusted logistic regression relative risk (RR); the grey shaded area represents the 95% confidence interval; text: *p*-value. (**B**) Sex-adjusted logistic regression relative risk of IgA seropositivity for the CTQ subscales identifies physical abuse as a key element of the overall CTQ score contributing to the risk of IgA seropositivity. Circles represent the relative risk (RR); error bars: 95% confidence interval; text: *p*-value. *, *p* < 0.05; **, *p* < 0.01; ***, *p* < 0.005.

In line with other recent studies [49], 69% of the total cohort reported no exposure to ELA, 19% of the participants were above the threshold in one category, 4% in two sub-categories, and <1% reported four or five different trauma types. We subsequently investigated the dose–response relationship of IgA seropositivity to the overall CTQ score. For all ELA exposure values CTQ >2, the RR was increased significantly, from 4.0- to 27-fold (*p* from 0.06 to 0.005; Figure 2A). When ELA exposure increased, the risk of IgA seropositivity increased in a similar manner (Spearman Correlation: 0.829, *p* = 0.058; from a CTQ score of 1.25 to 2.5) showing a clear dose–response relationship (Figure 2A). 

### 3.4. Physical Abuse Is the Predominant Driver of ELA

We examined the five subscales of the CTQ to identify the principal forms of ELA driving the association with IgA seropositivity. Using the subscales as continuous variables, physical abuse (PA) significantly raised the risk of IgA seropositivity by 4.38-fold (*p* = 0.009) (Figure 2B). The other CTQ subscales were not significant (*p*-value from 0.6 to 0.82). We concluded that the ELA plays a role in IgA seropositivity risk, and the largest contribution to this risk is from the physical abuse component.

### 3.5. Adult Trauma Is a Risk Factor for IgA Seropositivity

**Figure 3:**
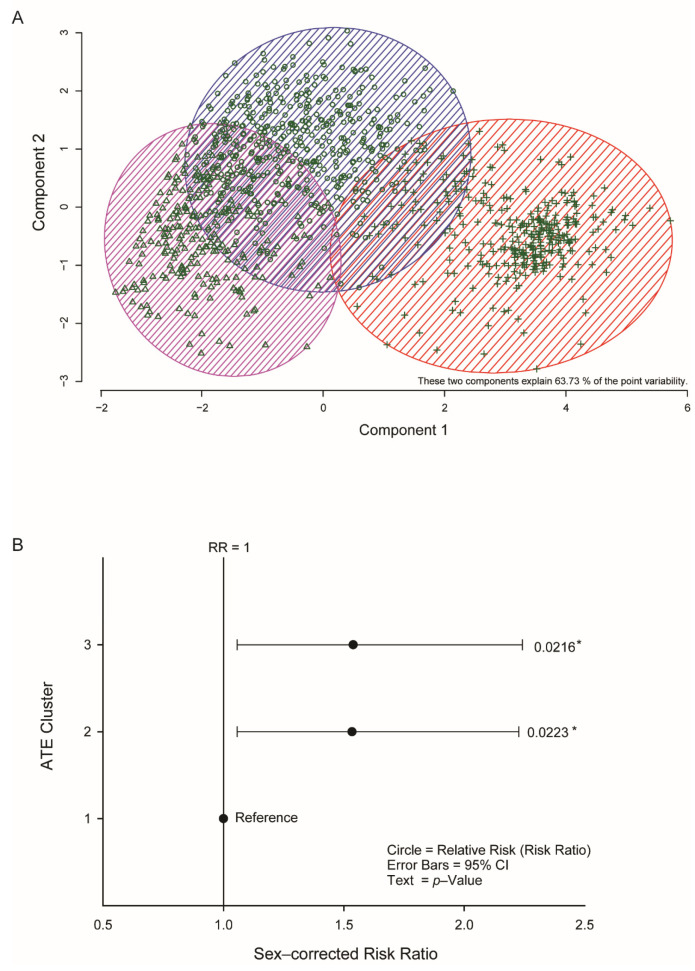
Exposure to adult traumatic events (ATE) is linked to IgA seropositivity in asymptomatic or oligosymptomatic individuals. (**A**) The two principal components after K-means clustering of the responses to the ATE questionnaire identified three clusters of responses. Circles (blue shaded area)—Cluster 1; Triangles (pink shaded area)—Cluster 2; Crosses (red shaded area)—Cluster 3. (**B**) Sex-adjusted relative risk CTQ subscales identified ATE clusters 2 and 3 (negative and positive salience, respectively) as having a similar effect on SARS-CoV-2 IgA seropositivity. Circles represent the relative risk (RR); error bars: 95% confidence interval; text: *p*-value. *, *p* < 0.05.

Measurement of adult trauma is complicated by the “salience” or the importance attached to the event [43] and the ATE questionnaire scored salience from −3 to 0 to +3 (negative experience, irrelevant, positive experience, respectively). Consequently, raw questionnaire data underwent k-means clustering to find patterns in the data. Three clear clusters were identified (Figure 3). Mean responses to the individual questions for the three clusters are included in the Supplementary Table S1. By inspection, it is clear that the three clusters can be interpreted as follows: cluster 1 had the lowest trauma; cluster 2 experienced trauma and gave it a negative salience; cluster 3 experienced trauma and gave it a positive salience. The crude RR of IgA seropositivity was significantly increased in clusters 2 and 3 (RR = 1.48, 95% CI:1.05–2.05, *p* = 0.023 and RR = 1.48, 95% CI:1.07–2.07, *p* = 0.0216, respectively; Figure 3B). We examined the risk of IgA seropositivity when ELA and ATE were both present (Supplementary Table S2). Previous studies have already demonstrated the impact of childhood traumatic events on adult life [17,24]. When ELA was present, ATE cluster 1 (reference) and 2 (*p* = 0.94 and 0.13) were not significant despite a high RR (from 1.07 to 7.53). Cluster 3 showed a significant result increase in RR (RR = 3; 95% CI 1.39–6.48; *p* = 0.035) with 40% IgA seropositive (4/10 participants). However, the numbers of participants in all three subcategories were low (range 1–10) rendering their interpretation unreliable. It is, however, safe to conclude that people exposed to ATE had an increased risk of being IgA seropositive regardless of their ELA experience.

### 3.6. Socioeconomic, Employment, and Life Covariates Do Not Influence IgA Seropositivity 

**Figure 4:**
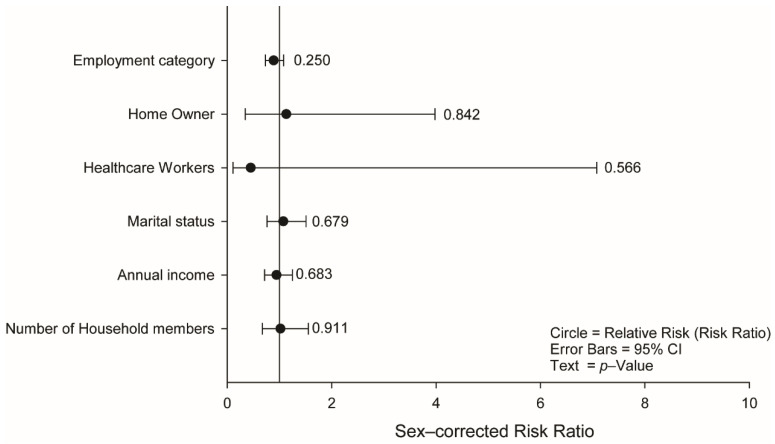
Socioeconomic covariates do not determine SARS-CoV-2 IgA seropositivity in asymptomatic or oligosymptomatic individuals. Sex-adjusted logistic regression relative risk of SARS-CoV-2 IgA seropositive due to current socioeconomic conditions. Circles represent the relative risk (RR); error bars: 95% confidence interval; text: *p*-value.

Given the important of asymptomatic carriers in viral transmission and their role in the COVID-19 pandemic, we examined the impact of socioeconomic parameters on IgA seropositivity (Figure 4) to see if any particular category had an increased exposure to SARS-CoV-2. Overall, there was no effect of any of the socioeconomic parameters including annual income, marital status, number of household members, employment category, or home ownership. In a secondary analysis these were dissected by category, confirming the numbers of participants in each category were sufficient and that there were no individual categories that were significantly associated with increased IgA seropositivity (Supplementary Table S3). Overall, these data highlight that in our study cohort the virus appeared to be circulating irrespective of socioeconomic context as recently suggested by [50], although this may be affected by the phase of the pandemic [51].

### 3.7. The Influence of ELA on Psychological States during Lockdown 

Although psychiatric comorbidities do not constitute a significant risk factor for exposure to SARS-CoV-2, exposure to ELA may not only influence their subsequent development, but may also have a significant effect on the psychological reaction to the lockdown containment measures. Prior exposure to ELA increased the relative risk of developing psychiatric disorders in our cohort (crude RR = 16.91, 95% CI: 6.09–46.91, *p* < 0.001; sex-adjusted RR = 11.47, 95% CI: 3.89–33.85, *p* < 0.001). At the first study visit, this link was clearly visible in the psychological questionnaires. 

**Figure 5:**
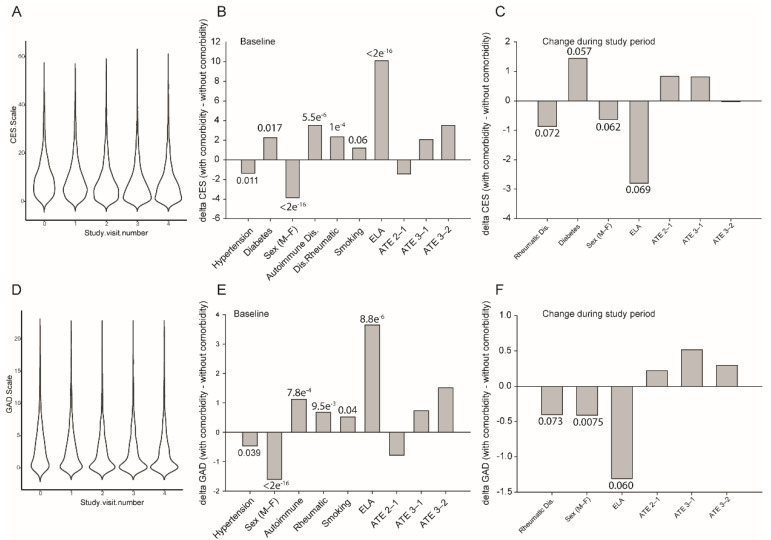
Psychological response to the containment measures during the study period in healthy, asymptomatic, and oligosymptomatic individuals. (**A**) Data density plot for the response to the Center for Epidemiologic Studies Depression Scale (CES-D) for the five experimental visits. (**B**) Significant group differences in CES-D score at the baseline CON-VINCE visit. Full statistical data are included in Supplementary Table S3. Data are from univariate ANOVA analysis, text above/below each bar is the ANOVA *p*-value. (**C**) Significant group differences in the change in CES-D score between the baseline and last CON-VINCE visit. Full statistical data are included in Supplementary Table S4. Data are from univariate ANOVA analysis, text above/below each bar is the ANOVA *p*-value. The full multiparameter ANOVA model is described in the Results Section. (**D**) Data density plot for the response to the Generalised Anxiety Disorder-7 (GAD-7) questionnaire for the five experimental visits. (**E**) Significant group differences in GAD-7 score at the baseline CON-VINCE visit. Full statistical data are included in Supplementary Table S4. Data are from univariate ANOVA analysis, text above/below each bar is the ANOVA *p*-value. (**F**) Significant group differences in the change in GAD-7 score between the baseline and last CON-VINCE visit. Full statistical data are included in Supplementary Table S4. Data are from univariate ANOVA analysis, text above/below each bar is the ANOVA *p*-value. The full multiparameter ANOVA model is described in the Results Section.

*CES (Depression):* The mean CES score for the complete cohort declined from 10.00 +/− 8.01 to 8.01 +/− 8.32 during this period (Wilcoxson test *p* < 2.2 × 10^−16^; Figure 5A). In univariate analyses, the baseline CES score was strongly influenced by multiple covariates (*p*-values from 0.017 to 2 × 10^−16^; Supplementary Table S3; Figure 5B). However, the change in CES score between inclusion and the end of the study period was dependent on the ATE cluster (*p* = 0.039) and ELA (*p* = 0.0217), with a trend towards significance for diabetes (*p* = 0.0574), sex (*p* = 0.062), and pre-existing rheumatic disease (*p* = 0.072) (Supplementary Table S4; Figure 5C). When these covariates were included in a multi-way-ANOVA (not shown), the change in CES score over the study period was significantly influenced by pre-existing rheumatic disease (main effect: F(1,1418) = 6.145, *p* = 0.0133), prior exposure to ELA (main effect: F(1,1418) = 9.814, *p* = 0.0018, and diabetes (main effect: F(1,1418) = 4.990, *p* = 0.0257). Confirming our prior hypothesis that diabetes may be linked to ELA [10], there was a significant interaction between comorbid diabetes and ELA on the change in CES score (interaction: F(1,1418) = 2.454, *p* = 0.117). Tukey post hoc analysis confirmed that prior exposure to ELA decreased the change in CES score by 12.2 points (95% CI: 2.10–22.35, *p* = 0.01) in participants with diabetes. A similar interaction was seen for ELA exposure, rheumatic diseases, and the change in CES score (interaction: F(1,1418) = 5.35, *p* = 0.0188). Tukey post hoc analysis confirmed that prior exposure to ELA increased the CES score by 9.04 points (95% CI: 1.83–16.27, *p* = 0.007) in participants with rheumatic disorders compared to those without. Similarly, participants with rheumatic disease had an increase in CES score over the study period of 8.72 points (95% CI: 0.25–17.19; *p* = 0.04).

*GAD (Anxiety)*: As for the CES, the baseline GAD scores depended on nine covariates (*p* values from 0.04 to 2 × 10^−16^; Supplementary Table S3; Figure 5E). However, the change in GAD score between inclusion and the end of the study period was dependent on sex (*p* = 0.0075), ELA (*p* = 0.061), ATE cluster (*p* = 0.062), age (*p* = 0.070), and pre-existing rheumatic disease (*p* = 0.073; Supplementary Table S4; Figure 5F). In a multi-way-ANOVA, the change in GAD score was significantly influenced by ELA exposure (main effect: F(1,1418) = 9.695, *p* = 0.0019), pre-existing rheumatic disease (main effect: F(1,1418) = 5.715, *p* = 0.016), and age (main effect: F(1,1418) = 1.328, *p* = 0.047). The only significant interaction was between ELA exposure and ATE (interaction: F(1,1418) = 5.705, *p* = 0.0035) (Supplementary Table S4).

*UCLA (Loneliness)*: Baseline UCLA scores depended on nine covariates (*p*-values from 5.85 × 10^−13^ to 0.061; Supplementary Table S3); however, in individual univariate analyses, the change in UCLA score over the study period was only dependent on exposure to ELA (F(1,1418) = 6.724, *p* = 0.0096).

*PSS (perceived stress)*: Baseline PSS scores depended on seven covariates (*p*-values from 5.5 × 10^−11^ to 0.037; Supplementary Table S3); however, in individual univariate analyses, the change in PSS score was dependent on a concurrent autoimmune (F(1,1436) = 9.072, *p* = 0.0026) or rheumatic disease (F(1,1400) = 3.856, *p* = 0.050). In a two-way ANOVA, the change in PSS score was only influenced by pre-existing autoimmune disease (main effect: F(1,14) = 9.399, *p* = 0.0022) (Supplementary Table S4).

## 4. Discussion

Using the CON-VINCE cohort of healthy, asymptomatic, and oligosymptomatic individuals, we were able to demonstrate, at the population level, that exposure to a life history of traumatic events significantly increased the risk of SARS-CoV-2 IgA seropositivity, as did gender and smoking. Furthermore, a prior history of adversity was a key driver in the psychological reaction during the period of strict containment measures.

There is now a plethora of data available on the demographics of SARS-CoV-2 patients with active symptomatic disease in both the community and hospital situation [52,53,54,55,56]. Although we saw a clear sex bias in IgA seropositivity, an increase associated with other cases in the family home, and a decrease in seropositivity in active smokers, our data present a very different picture to COVID-19 patient cohorts. In our logistic regression relative risk models, only sex remained as a statistically significant covariate. Our initial statistical model suggested that neither age nor BMI were significant covariates. We confirmed both results in a secondary analysis, with all age and BMI categories having similar, statistically non-significant, RRs. Together with the socioeconomic data, these data indicate a generalised circulation of the SARS-CoV-2 throughout the population. A prior cancer diagnosis was a protective factor. Although this warrants further investigation, the most probably interpretation of this is a “conscientious phenotype”. This may be true for household contacts too. We observed a non-significant 37% increase in RR from household contacts, higher than that which was recently reported, although we had less power to detect such associations [57]. As recently reported, there is a public under-appreciation of the importance of barriers. It is possible that behavioural modifications and awareness of the importance of health behaviours in this population may underlie stricter adherence to social distancing, facemask usage, and disinfectant gel usage, reducing the relative risk [58]. Similarly, the RR did not increase with the number of household members; however, in the context of significantly reduced social contact (i.e., lockdown) this result may not be surprising. This contrasts with the situation seen in COVID-19 patients. It is important to differentiate the risk of exposure from disease severity. As we have previously highlighted [10], data from Chicago clearly identified increased mortality in ethnic minorities [59]. They represented up to 70% of the overall COVID-19 deaths, and as the local population in lower socioeconomic classes increased, the local mortality rate increases significantly [14]. The authors ascribed this to both exposures from poverty and over-populated housing, as well as severity from pre-existing comorbidities such as type 1 and 2 diabetes or cardiovascular disease [7]. We previously interpreted these reports as indicating the effect of current SES and environment conditions on SARS-CoV-2 morbidity and infection rates [10]. The present data suggest that it is not an effect of SES or the environment that leads to higher rates, but rather, these rates are representative of an underlying exposure to traumatic life events that changes the risk of exposure. Our prior interpretation may be partly correct, however, since SES and ELA correlate closely [60,61]. Our early-life data are almost unique in that they are from a cohort that is statistically representative of a national population. The rate of exposure to ELA concords with the only similar data available [49] confirm that our data will not be unique to Luxembourg, but representative of a wider European population. Indeed, recent data from the UK Biobank cohort confirmed the importance of this early-life period, as having been breastfed ~70 years ago still provided protection, whilst maternal smoking during gestation significantly increased the risk of SARS-CoV-2 infection during the pandemic [62].

Our data highlight two interesting risk factors: smoking and exposure to psychosocial adversity. Our observation that current smokers or their partners have a reduced risk of SARS-CoV-2 seropositivity agrees with clinical reports and a recent meta-analysis that smokers are underrepresented, by up to a factor of 10-fold in hospitalised cohorts [63]. This is, however, counterbalanced by reports that upon SARS-CoV-2 infection, smoking may increase the overall severity and progression of COVID-19 [64]. Mechanistically, this would appear to pass through changes in levels of ACE2, although the data are somewhat contradictory with both smoking-induced increases [65] and decreases [66] in ACE2 levels reported. This somewhat counter-intuitive result in smokers has to be treated with caution, as it may be due to a social desirability bias. Underreporting of smoking, alcohol, or drug use remains frequent, although internet-based self-reported data collection goes some way to alleviate this bias [67]. It is possible that this represents a similar “conscientious phenotype”, with smokers taking more care as they perceive a higher risk, although there are no data to confirm this. Our observation that psychosocial adversity increases the risk of SARS-CoV-2 seropositivity concurs with the data from the 1970s [68] that social adversity is linked to more frequent infections as well as non-communicable diseases [69]. Furthermore, our data follow the same direction as a series of reports over the last few years that highlight the exaggerated effect of early-life adversity on adult immune function [70,71]. Adverse social conditions appear to be embedded as long-term functional changes in the immune system. The available data suggest that as little as 4 months of exposure to ELA can change the immune response up to ~24 years later [28,72,73,74]. Such exposure drives the accumulation of senescent immune cells that not only appear to have a decreased capacity to proliferate, but also, their responsiveness to subsequent bacterial or viral stimuli is reduced [28,30]. This association particularly strong for the senescent CD8+ CD57+ TEMRA cells that lose the ability to mount an effective immune response to a new infection, a finding that has been independently replicated [28,30,74]. Early-life social adversity also acts by enhancing the expression of inflammatory and T-lymphocyte activation genes, while concurrently reducing the expression of type I IFN-mediated innate antiviral response genes, as well as other pathogen-specific innate antimicrobial response genes [72]. These are patterns of altered gene expression that remain lifelong [72]. This specific gene expression pattern is termed the “conserved transcriptional response to adversity (CTRA)” and has been reported in many human observational studies of adversity [75,76,77,78,79,80,81,82]. The CTRA is most strongly induced by adversity in early life, corresponding to the postnatal period during which the immature immune system develops and starts maturing [72]. In a manner similar to the functional changes in the immune cells, this transcriptome remodelling persists, affecting the immune responses to pathogens or allergens encountered many years later [76,83,84]. 

Our data highlight the negative effect of ELA, ATE, and pre-existing autoimmune or rheumatic disorders on depression and anxiety levels during the lockdown period. Such containment measures have been associated with negative mental health outcomes, but a perception of performing essential work, receiving kindness, and community connectedness were associated with positive mental health outcomes [85]. There is a tight link between both autoimmune and rheumatic diseases and hypothalamus–pituitary–adrenal (HPA) axis functioning, with exaggerated responses to daily stressors [86,87]. Similarly, exposure to ELA or ATE affects HPA axis functioning [88]. This was seen after ELA in cohorts 10–12 years post ELA [89] or ~24 years after ELA [90]. Prior exposure to ELA has also been linked to stress-induced negative moods and emotions [89]. As such, the changes in the CES and GAD questionnaire appear to be consistent with the existing literature.

To evaluate the risk of exposure and seroconversion to SARS-CoV-2, we analysed IgA in preference to IgG or IgM, as levels of the latter are not only significantly lower in asymptomatic SARS-CoV-2-positive individuals than in COVID-19 patients [9], whilst IgA levels are higher and seroconversion occurs within 2 days of infection compared with up to 32 days for IgG and IgM [91]. Our data confirmed this, as we only had 39 IgG-positive cohort members giving a non-representative and non-significant conclusion, compared to 209 IgA-positive participants. Previously, we reported the specificity and sensitivity of the IgA and IgG ELISAs using hospitalised COVID-19 patients and a pre-pandemic cohort sera. The specificity of the IgA ELISA used was lower than for IgG (89.2% vs. 97.8%);however, it was more sensitive (92.9% vs. 85.7%) [2]. The large discrepancy between the number of IgA- and IgG-positive participants may be in part due to the lower specificity of the IgA ELISA; however, they are more likely to come from the sequential nature of the immune response, since IgA appears sooner than IgG [92], and is a stronger neutraliser of SARS-CoV-2 than either IgM or IgG [93]. Furthermore, IgA would also appear to be more relevant in mild infections as SARS-CoV-2 infection is, in principal, restricted to the upper respiratory tract, with the infection spreading to the lower respiratory tract only in more severe cases [31]. As reviewed by Russell et al. (2020), it would naturally be expected that in mild cases the primary immune response originates from the mucosal immune system, particularly from the nasopharynx-associated lymphoid tissue (NALT) [32]. The NALT is an inductive site for the mucosal immune system, and it includes the nasal epithelium as well as the adenoids and the tonsils [32]. It has been proposed that the bronchus-associated lymphoid tissue (BALT) that is normally found to form after infection, particularly in adolescents and children [94], may underlie the increased resistance of children and adolescents to the COVID-19 disease. The NALT and GALT generate almost exclusively an IgA response from mucosal B cells that locally differentiate into IgA-secreting plasma cells, although a small number of IgG-producing B cells are induced in NALT tissues such as the tonsils, producing detectable levels of IgG (and IgM) in the circulation [95]. Unfortunately, there has been a preponderance to study circulating IgG and IgM levels rather than the IgA levels [96,97,98]. However, in mild infections, IgM and IgG may only be effective if they can reach the infected upper respiratory tract mucosae, but they are not readily transported to mucosal surfaces [99]. Indeed, serious COVID-19 infection is associated with infections in the lower rather than the upper respiratory tract, particularly in the terminal airways. Here, IgG is the predominant class, and the intensely inflammatory nature of IgG induces severe COVID-19 infection through inflammation, complement activation, and induction of phagocytosis by, e.g., macrophages, neutrophils, and the activation of the cellular immune response, including CD4+ and cytotoxic CD8+ T cells that cannot, by their nature, prevent infection. Their role being to destroy infected cells to reduce the risk of the infection propagating, with a high cost. The best data available show that IgG and IgM levels do not accurately reflect prior PCR-confirmed mild infection, or patients did not seroconvert [100], and by ignoring IgA, the seroprevelance is significantly underestimated [101]. The most commonly used anti-nucleocapsid IgG ELISA identified 40/42 (95.2%) of severe hospitalized cases as seropositive, but in mild, non-hospitalised cases, only identified 539/1134 (47.5%) cases were seropositive; furthermore, the anti-nucleocapsid IgM assay failed to detect 95% of these milder cases [100]. Contrastingly, when the pan-Ig test, including IgA, was used, seroconversion was detected in >90% of the mild cases leading to the conclusion that measuring IgA is essential [93,100,102,103,104]. Based on these observations and on the lack of sufficient number of IgM participants in our cohort, we conclude that IgA is more relevant for our population survey than either IgG or IgM.

We calculated the seropositivity risk using the relative risk (risk ratio). Although there are multiple models available (Cox regression, relative risk, odds ratio), we considered participants to be seropositive for either IgA or IgG if, during the study period, they had one or more positive serology results, and we did not consider the time at which they became positive during the study, negating the use of Cox’s hazard ratio. As approximately 13% of the CON-VINCE cohort were IgA seropositive [2], the “rare disease assumption” that the odds ratio is similar to the relative risk when the outcome incidence is low did not hold true [92]. Furthermore, as the cohort is statistically representative of the whole country that was in lockdown during the study period, the 199 IgA seropositive participants was a genuine representation of the silent spreader population within the country at that time. As such, we calculated the RR, using sex-corrected logistic regression models, as the most appropriate measure in our cohort. Our cohort was recruited and repeatedly sampled over a 10-week period at the tail-end of the first epidemic wave in Luxembourg [2]. Although IgA has a plasma half-life of around 3–5 days, there is evidence that circulating antibodies to the related SARS-CoV-1 and MERS-CoV remain detectable for >12 months [105,106], and the more stable IgG remains detectable for 24–36 months after SARS-CoV-1 or MERS-CoV infection [107,108]. Recent data from Iceland suggests that >100 days post exposure, asymptomatic individuals still have detectable IgA and IgG levels [109].

As such, we are confident that the serology results obtained during the study period are reflective of the exposure during the entire period from the first case in Luxembourg on 29 February 2020, through the start of recruitment and sampling on 15 April 2020, to the end of the study on 5 May 2020. In the event of our increased IgA seropositivity being due to cross-reactivity with seasonal coronaviridae, the fundamental observation remains. A life history of traumatic events increases exposure to either SARS-CoV-2 or other coronaviridae.

Asymptomatic individuals represent a reservoir of virus that is proving to be an obstacle in managing the COVID-19 pandemic. In this study, we clearly identified the demographics of asymptomatic SARS-CoV-2-infected individuals. This profile was unique in that there were no underlying factors that predisposed individuals to being SARS-CoV-2 seropositive, nor were there factors that predisposed individuals to having a mild disease course. As recently reported, age was not a factor in mild SARS-CoV-2 infections [91,110]. Furthermore, there was no effect of any of the socioeconomic factors investigated; however, a prior exposure to traumatic life events appears to be one of the strongest predictors, along with sex, smoking, and having a SARS-CoV-2-positive family member, for being exposed to SARS-CoV-2 and becoming seropositive. Additionally, a life history of traumatic events or concurrent autoimmune or rheumatic disease were associated with a worse evolution of anxiety and depressive symptoms throughout the lockdown period.

The clear connection between SARS-CoV-2 seropositivity and a life history of adversity is particularly promising for future studies. There are, however, several areas that need to be investigated to take these results further. The role of the mucosal immune system, in particular IgA, needs to be clarified. Furthermore, our observations need to be expanded to more severe forms of COVID-19, where the well-established cellular immune deficiencies induced by ELA may also play an important role. While our data remain preliminary because they were taken over a very short period at the start of the pandemic, it will be essential to follow our CON-VINCE cohort over a longer period to see whether our epidemiological link between psychosocial adversity is retained with time, and whether over a longer period the IgA response has matured into an IgG response.

## Figures and Tables

**Table 1:** Categorical breakdowns of key demographic covariates.

Age Category	Total	Female/Male	IgA Positive	RR (95%CI; *p*-Value)
18–29	150	90/59	21 (14%)	1 (–)
30–39	260	133/128	42 (16%)	1.15 (0.7–1.9; 0.67)
40–49	325	177/147	43 (13%)	0.94 (0.58–1.53; 0.88)
50–59	297	156/142	34 (13%)	0.82 (0.49–1.36; 0.45)
60–69	272	150/123	34 (11%)	0.89 (0.54–1.48; 0.65)
70–79	154	49/107	24 (15.5%)	1.11 (0.65–1.91; 0.75)
BMI Category				
Underweight	32	26/6	5	1.12 (0.49–2.58; 0.79)
Normal	614	333/281	85	1 (–)
Overweight	493	228/265	60	0.87 (0.65–1.20; 0.42)
Obese	334	170/167	49	1.05 (0.76–1.46; 0.77)
Smoking Category				
Never smoked	796	444/354	120 (15.1%)	1 (–)
Live with smoker	439	180/260	58 (13.2%)	0.89 (0.66–1.17; 0.40)
Ex-smoker	38	19/19	6 (15.7%)	1.05 (0.49–2.22; 0.82)
Current smoker	201	114/86	15 (7.4%)	0.50 (0.30–0.83; 0.004)

**Table 2:** Number of participants with each of the participant-reported comorbidities.

Disease Categories	Case Numbers	Female/Male	*p*-Value (Chi^2^)	IgA Positive
Cardiac	53	17/36	0.009058	7 (13.2%)
Hypertension	269	113/156	0.008748	36 (13.4%)
Pulmonary	35	17/18	0.8658	6 (17%)
Liver	36	17/19	0.7389	2 (5%)
Kidney	13	7/6	0.7815	2 (15%)
Rheumatological	199	121/78	0.002302	20 (10.1%)
Autoimmune	115	90/25	1.35 × 10^−9^	17 (14.7%)
HIV	6	1/5	0.1025	1 (16%)
Cancer	85	38/47	0.329	12 (14.1%)
Haematological	19	11/8	0.4913	1 (5%)
Malnourished	4	2/2	1	2 (50%)
Diabetes (I + II)	76	32/44	0.1687	9 (11.8%)
Transplant	7	3/4	0.7055	1 (14.3%)
Psychiatric	69	45/24	0.01529	7 (10.1%)
Other	0	0/0	n/a	0 (0%)
CTQ >2	18	17/1	4.56 × 10^−10^	6 (33.3%)

## Data Availability

Data from the CON-VINCE study are available upon reasonable request and after approval from the CON-VINCE study executive committee, chaired by R.K. The reduced dataset reported in this manuscript is also available upon reasonable request to either J.D.T. or R.K.

## Supplementary Materials

The following are available online at https://www.mdpi.com/article/10.3390/jcm10102159/s1, Table S1: mean ATQ responses to the individual questions for the three clusters, Table S2: risk of IgA seropositivity when ELA and ATE are both present, Table S3: univariate analyses of the baseline for psychological scales, Table S4: univariate analyses of the delta between V0 and V4 for psychological scales.
