# Supplementary material for: Adverse Life Trajectories Are a Risk Factor for SARS-CoV-2 IgA Seropositivity
Source: J Clin Med. 2021 May 17;10(10):2159. doi: 10.3390/jcm10102159 (PMC8157140; doi:10.3390/jcm10102159)
Supplement: Supplementary file 1 [file jcm-10-02159-s001.zip › IEE35 Holuka supplementary v2.pdf]

Journal of
Clinical Medicine

MDPI

Article

Adverse life trajectories are a risk factor for SARS-CoV-2 IgA seropositivity

Cyrielle Holuka[1,2], Chantal J. Snoeck[3], Sophie B. Mériaux[1], Markus Ollert[4,5], Rejko Kruger[6,7], and Jonathan D. Turner[1,*] on behalf of the CON-VINCE Study Group *

[1] Immune Endocrine Epigenetics Research Group, Department of Infection and Immunity, Luxembourg Institute of Health, Esch-sur-Alzette, Luxembourg
[2] Faculty of Science, University of Luxembourg, Belval, Luxembourg
[3] Clinical and Applied Virology Group, Department of Infection and Immunity, Luxembourg Institute of Health, Esch-sur-Alzette, Luxembourg
[4] Allergy and Clinical Immunology, Department of Infection and Immunity, Luxembourg Institute of Health, 29, rue Henri Koch, L-4354 Esch-sur-Alzette, Luxembourg
[5] Department of Dermatology and Allergy Center, Odense Research Center for Anaphylaxis, University of Southern Denmark, Odense C, Denmark
[6] Transversal Translational Medicine, Luxembourg Institute of Health (LIH), Strassen, Luxembourg.
[7] LCSB, Luxembourg Centre for Systems Biomedicine, University of Luxembourg, Esch-Sur-Alzette, Luxembourg
* Correspondence: jonathan.turner@lih.lu Tel.: +352 2697 0629

Supplementary material

Contents:

Supplementary Table 1 p2
Supplementary Table 2 p3
Supplementary Table 3 p4
Supplementary Table 4 p6

Supplementary table 1

	Cluster			
How did the following life events affect you :	1	2	3	p-value
Q1 Separation from a partner:	-1.22 ± 1.97)	-2.00 ± 1.71	0.599 ± 1.21	<2e-16
Q2 Separation of your parents:	-1.64 ± 1.84	-2.31 ± 1.47	0.636 ± 1.14	<2e-16
Q3 Your parents starting new relationships:	-2.42 ± 1.34	-2.72 ± 0.972	0.444 ± 1.32	<2e-16
Q4 Money worries: Major life events shape your overall health.	-0.568 ± 1.75	-2.57 ± 1.15	0.704 ± 0.798	<2e-16
Q5 Money worries for other family members:	-0.726 ± 1.81	-2.66 ± 1.05	0.688 ± 0.712	<2e-16
Q6 Accident / illness:	0.0912 ± 1.45	-2.54 ± 1.16	0.736 ± 0.811	<2e-16
Q7 Accident / illness of a family members	0.430 ± 1.24	-1.97 ± 1.68	0.710 ± 0.862	<2e-16
Q8 Losing your job:	-2.52 ± 1.25	-2.84 ± 0.731	0.723 ± 1.00	<2e-16
Q9 Job loss of a close family member:	-2.21 ± 1.50	-2.80 ± 0.801	0.789 ± 0.826	<2e-16
Q10 Being a victim of violence:	-2.67 ± 1.04	-2.86 ± 0.709	0.717 ± 1.16	<2e-16
Q11 A family member being a victim violence:	-2.69 ± 1.03	-2.84 ± 0.726	0.697 ± 1.16	<2e-16
Q12 Death of a family member:	0.441 ± 1.48	-1.48 ± 1.91	0.845 ± 1.07	<2e-16

Supplementary table 1: mean responses to the individual questions for the three clusters.

Answers to individual question for each cluster are represented in the table.

Supplementary Table 2

	number	Female/male (Chi2)	IgA positive	RR (95%CI; p-value)
CTQ>2	18	17/1	6 (33%)	6.51<br>(0.78- 49.27; 0.060)
CTQ+ATE1	7	6/1 (0.0027)	1 (14%)	1.07<br>(0.06 - 6.33; 0.948)
CTQ+ATE2	1	1/0 (0.392)	1 (100%)	7.53<br>(6.60 - 8.59; 0.133)
CTQ+ATE3	10	4/6 (1.38e-06)	4 (40%)	3.00<br>(1.39 - 6.48; 0.035)

Supplementary table 2: risk of IgA seropositivity when ELA and ATE are both present. For each cluster, ratio female/male and statistics of IgA positivity are given in the table.

Supplementary Table 3

Comorbitidies	CES	GAD	UCLA	PSS	BRS
Mild liver	F(1, 1439) = 1,846, p= 0,174	F(1, 1439)= 0,164, p= 0,685	F(1, 1439)= 1,151, p= 0,698	F(1, 1439)= 0,062, p= 0,803	F(1, 1439)= 1,417, p= 0,739
Hypertension	F(1, 1454)= 6,452, p = 0,0112	F(1, 1454)= 4,244, p= 0,0396	F(1, 1454)= 4,561, p= 0,0329	F(1, 1454)= 2,89, p= 0,0894	F(1, 1454)= 1,211, p= 0,271
Chronic kidney	F(1, 1461)= 0,142, p = 0,707	F(1, 1476)= 0,617, p= 0, 432	F(1, 1461)= 2,108, p= 0,147	F(1, 1461)= 2,067, p= 0,151	F(1, 1461)= 0,506, p= 0,477
Chronic pulmonary	F(1, 1458)= 0,3,519, p = 0,609	F(1, 1458)= 0,702, p= 0,402	F(1, 1458)= 0,57, p= 0,45	F(1, 1458)= 0,655, p= 0,419	F(1, 1458)= 2.697, p= 0,101
Diabete	F(1,1444) = 5, 742, p= 0,0167	F(1, 1444)= 0,006, p= 0,941	F(1, 1444)= 1, 606, p= 0,205	F(1, 1444)= 0,005, p= 0,944	F(1, 1444)= 0,053, p= 0,817
Age Category	F(5, 1455)= 10,4, p= 7.99e-10	F(5, 1455)= 7,689, p= 3,8e-07	F(5, 1455)= 6,608, p= 4,31e-06	F(5, 1410)= 6,123, p= 1.27e-05	F(5, 1455)= 1,332, p=0,248
BMI Category	F(3,1472)=3,315, p= 0,0193	F(3, 1472)= 3,215, p= 0,0221	F(3, 1472)= 3,315, p= 0,0193	F(3, 1472)= 1,352, p= 0,256	F(3, 1472)= 1,345, p= 0,258
Sex	F(1, 1472)= 91,61, p= <2e-16	F(1, 1472)= 84,9, p= <2e-16	F(1, 1472)= 43,63, p= 5,53e-11	F(1, 1472)= 25, 16, p= 5,91e-07	F(1, 1472)= 4,858, p= 0,0277
Autoimmune	F(1, 1436)= 20,83, p= 5,45e-06	F(1, 1436)= 11,33, p= 0,000784	F(1, 1436)= 4, 354, p= 0,0371	F(1, 1436)= 0,419, p= 0,517	F(1, 1436)= 2,137, p= 0,144
Cancer	F(1, 1461)=0,927, p= 0,336	F(1, 1461)= 2,48, p= 0,115	F(1, 1461)= 0,295, p= 0,587	F(1, 1461)= 3,525, p= 0,0607	F(1, 1461)= 0,298, p= 0,585
Hematologic	F(1, 1466)=1,113, p= 0,292	F(1, 1466)= 0,311, p= 0,577	F(1, 1466)= 0,037, p= 0,848	F(1, 1466)= 0,559, p= 0,455	F(1, 1466)= 1,765, p= 0,184
HIV	F(1,1464)=0,137, p= 0,712	F(1, 1464)= 0,02, p= 0,887	F(1,1464)= 7,953, p= 0,00487	F(1, 1464)= 3,278, p= 0,0704	F(1, 1464)= 0,272, p= 0,602
Malnourished	F(1, 1463)= 15,79, p= 7.43e-05	F(1, 1463)= 21,14, p= 4,63e-06	F(1, 1463)= 14,35, p= 0,000158	F(1, 1463)= 8,825, p= 0,00302	F(1, 1463)= 2,112, p= 0,146
Organ transplant	F(1,1474)= 0,02, p= 0,886	F(1, 1474)= 0,05, p= 0,823	F(1, 1474)= 0,126, p= 0,722	F(1, 1474)= 2,98, p= 0,0845	F(1, 1474)= 0,337, p= 0,562
Rheumatic	F(1, 1400)= 15,22, p= 1e-04	F(1, 1400)= 6,748, p= 0,00949	F(1, 1400)= 2,796, p= 0,0947	F(1, 1400)= 0,037, p=0,847	F(1, 1400)= 0,231, p= 0,631
Alcohol	F(1, 1473)= 3,69, p= 0,0549	F(1, 1473)= 0,162, p= 0,687	F(1, 1473)= 0,927, p= 0,336	F(1, 1473) = 2,863, p= 0,0908	F(1, 1473)= 5,949, p= 0,0148
Smoking	F(3, 1472)= 2,461, p= 0,0611	F(3, 1472)= 2,749, p= 0,0416	F(3, 1472)= 1,481, p= 0,218	F(3, 1472)= 3,788, p= 0,0101	F(3, 1472)= 1,209, p= 0,305
Cardiac	F(1, 1429)= 0,53, p= 0,467	F(1, 1429)= 0,017, p= 0,897	F(1, 1429)=0,165, p= 0,684	F(1, 1429)= 0,3, p= 0,584	F(1, 1429)= 0,006, p= 0,938

CTQ 2	F(1, 1420)= 35,99, p= 2,51e-09	F(1, 1420)= 24,95, p= 6,61e-07	F(1, 1420)= 17,58, p= 2,93e-05	F(1, 1420)= 17,59, p= 2,9e-05	F(1, 1420)= 1,401, p= 0,237
Psychiatric	F(1, 1453)= 108,9, p= <2e-16	F(1, 1453)= 95,56, p= <2e-16	F(1, 1453)= 15,24, p= 9,88e-05	F(1,1453)= 52,85, p= 5,85e-13	F(1, 1453)= 43,23, p= 6.78e-11
ATE	F(2, 1473)= 23,59, p= 9,77e-12	F(2, 1473)= 25,57, p=1,21e-11	F(2, 1473)= 6,081, p= 0,00234	F(2, 1473)= 11,15, p= 1,56e-05	F(2, 1473)= 5,15, p= 0,00591
BRS	F(1, 1474)= 72,69, P<2e-16	F(1, 1474)= 67,77, P= 4e-16	F(1, 1474)= 14, 3, P= 1,6e-4	F(1, 1474)= 28,23, P= 1,24e-7	N/A

Supplementary table 3: univariate analyses of the baseline for psychological scales. All statistics, at the beginning of the study, for each psychological questionnaire associated to comorbidities are given in the table.

Supplementary Table 4

Comorbitidies	CES	GAD	UCLA	PSS
Mild liver	F(1, 1439)= 0,298, p= 0,585	F(1, 1439)= 0,047, p= 0,828	F(1, 1439)= 0,001, p= 0,98	F(1, 1439)= 0,008, p= 0,929
Hypertension	F(1, 1454)= 0,147, p= 0,701	F(1, 1454)= 0,136, p= 0,713	F(1, 1454)= 0,237, p= 0,626	F(1, 1454)= 1,667, p= 0,197
Chronic kidney	F(1, 1461)= 0,731, p= 0,393	F(1, 1461)= 1,649, p= 0,199	F(1, 1461)= 1,032, p= 0,31	F(1, 1461)= 2,94, p= 0,0866
Chronic pulmonary	F(1, 1458)= 0,224, p= 0,637	F(1, 1458)= 0329, p= 0,566	F(1, 1458)= 0,031, p= 0,859	F(1, 1458)= 0,422, p= 0,512
Diabete	F(1, 1444)= 3,615, p= 0,0574	F(1, 1444)= 0,004, p= 0,951	F(1, 1444)= 0,145, p= 0,703	F(1, 1444)= 0,224, p= 0,636
Age Category	F(5, 1455)= 2,037, p= 0,0708	F(5, 1455)= 2,042, p= 0,0701	F(5, 1455)= 0,423, p= 0,833	F(5, 1455)= 2,374, p=0,0371
BMI Category	F(3, 1472)= 0,412, p= 0,744	F(3, 1472)= 0,029, p= 0,993	F(3, 1472)= 0,698, p= 0,553	F(3, 1472)= 1,16, p= 0,324
Sex	F(1, 1472)= 3,485, p= 0,0621	F(1, 1472)= 7,169, p= 0,0075	F(1, 1472)= 0,042, p= 0,838	F(1, 1472)= 0,794, p= 0,373
Autoimmune	F(1, 1436)= 0,134, p= 0,715	F(1, 1436)= 0,012, p= 0,911	F(1, 1436)= 0,337, p= 0,562	F(1, 1436)= 9,072, p= 0,00264
Cancer	F(1, 1461)= 0,305, p= 0,581	F(1, 1461)= 0,339, p= 0,561	F(1, 1461)= 0,174, 0,676	F(1, 1461)= 0,176, p= 0,675
Hematologic	F(1, 1466)= 0,021, p= 0,886	F(1, 1466) = 0,021, p= 0,885	F(1, 1466)= 0,234, p= 0,629	F(1, 1466)= 0,057, p= 0,811
HIV	F(1, 1464)= 0,036, p= 0,85	F(1, 1464)= 0,02, p= 0,887	F(1, 1464)= 0,834, p= 0,361	F(1, 1464)= 0,68, p= 0,41
Malnourished	F(1, 1463)= 1,534, p= 0,216	F(1, 1463)= 0,816, p= 0,366	F(1, 1463)= 0,002, p= 0,966	F(1, 1463)= 0,127, p= 0,722
Organ transplant	F(1, 1474)= 2,868, p= 0,0906	F(1, 1474)= 2,662, p= 0,103	F(1, 1474)= 1,988, p= 0,159	F(1, 1474)= 0,497, p= 0,481
Rheumatic	F(1, 1400)= 3,247, p= 0,0718	F(1, 1400)= 3,226, p= 0,0727	F(1, 1400)= 1,298, p=0,255	F(1, 1400)= 3,856, p= 0,0498
Alcohol	F(1, 1473)= 0,049, p= 0,826	F(1, 1473)= 0,533, p= 0,465	F(1, 1473)= 1,452, p= 0,228	F(1, 1473)= 0,04, p= 0,841
Smoking	F(3, 1472)= 1,999, p= 0,112	F(3, 1472)= 0,476, p= 0,699	F(3, 1472)= 0,346, p= 0,792	F(3, 1472)= 0,1, p= 0,96
Cardiac	F(1, 1429)= 0,021, p= 0,886	F(1, 1429)= 0,047, p= 0,828	F(1, 1429)= 0,311, p= 0,577	F(1, 1429)= 0,73, p= 0,393
CTQ 2	F(1, 1420)= 5,28, p= 0,0217	F(1, 1420)= 6,147, p= 0,0133	F(1, 1420)= 3,849, p= 0,05	F(1, 1420)= 4,77, p= 0,0291

Psychiatric	F(1, 1473)= 0,167, p= 0,683	F(1, 1453)= 1,122, p= 0,29	F(1, 1453)= 0,17, p= 0,68	F(1, 1453)= 0,338, p= 0,561
ATE	F(2, 1473)= 3,307, p= 0,0369	F(2, 1473)= 5,262, p= 0,00528	F(2, 1473)= 1,219, p= 0,296	F(2, 1474)= 3,391, p= 0,0339
BRS	F(1, 1474)= 0,423, P= 0,525	F(1, 1474)= 0,478, P= 0,486	F(1, 1474)= 2,898, P= 0,0889	F(1, 1474)= 28,23, P= 3,25e-3

Supplementary table 4: univariate analyses of the delta between V0 and V4 for psychological scales

All statistics, between the beginning and the end of the study, for each psychological questionnaire associated to comorbidities are given in the table.
